# Coordination dynamics of back-and-forth movement among expert performers: interaction in the battle scene of breaking

**DOI:** 10.3389/fpsyg.2025.1441378

**Published:** 2025-07-08

**Authors:** Daichi Shimizu, Takeshi Okada

**Affiliations:** ^1^Graduate School of Human Development and Environment, Kobe University, Nada-ku, Hyogo, Japan; ^2^Graduate School of Education, The University of Tokyo, Bunkyo-ku, Tokyo, Japan

**Keywords:** performing arts, interpersonal coordination, competitive context, breaking, back-and-forth movement, relative distance, relative phase

## Abstract

Complex interactions are central to the performing arts. While recent studies have explored these dynamics through synchronization and coordination theories, they have mainly focused on collaborative contexts. In contrast, genres like jazz sessions and breaking battles involve active competition, where performers seek to outshine one another. Although prior research has identified patterns like anti-phase synchronization in such settings, coordination across expressive channels and differences from sports interactions remain underexplored. To address this gap, the present study had two objectives: first, to investigate coordination through back-and-forth movements during breaking battles, and second, to compare these patterns with those observed in interpersonal sports. We conducted an experimental study simulating a battle scene with expert break dancers, examining how they coordinated their movements and managed relative distances. The results revealed two key findings: (1) dancers maintained close distances (~1.0 m) while coordinating through anti-phase synchronization (−180° to −160° and 160° to 180° relative phases), with coordination patterns shifting dynamically—from leader-follower relationships to anti-phase and then in-phase synchronization—depending on context and time; and (2) such time- and context-dependent coordination dynamics were unique to the performing arts and not observed in interpersonal sports. This study highlights the distinctive nature of context-sensitive, multi-channel interpersonal coordination in competitive performing arts.

## 1 Introduction

In the domain of the performing arts, which encompasses dance, theater, and musical performance, performers actively interact with each other to deliver compelling performances (Bailey, 1980; Merker et al., 2015). Both some performers and researchers have described this interaction as a fundamental aspect of the performing arts (Bailey, 1980), as the complex dynamics between performers captivate the audience's attention. Moreover, several theories suggested that such interaction plays a crucial role in human society (Fitch, 2006; Kirschner and Tomasello, 2010; Merker et al., 2009; Ravignani et al., 2014), as it fosters and strengthens social bonds, thereby contributing to the development and maintenance of communities. Given that humans benefit from living in large social groups (Dunbar, 1998), musical performances and dance have become widespread across all cultures and societies. Recent studies have indicated that participating in musical and dance activities can enhance interpersonal relationships, as individuals experience a sense of connection and cohesion through shared performance (Kirschner and Tomasello, 2010; Weinstein et al., 2016; Wiltermuth and Heath, 2009)

In recent years, several researchers have sought to investigate the complex interaction among performers by applying frameworks of synchronization and coordination (e.g., Keller et al., 2014; Ravignani et al., 2014; Walton et al., 2015, 2018; Washburn et al., 2014). Synchronization phenomena have been studied across various fields, including physics, biology, and psychology. They investigated the behavior matching of multiple objects, insects, or people, such as the swinging of clock pendulums, the flickering of firefly lights, and audience applause (Buck and Buck, 1966; Huygens, 1665; Jones, 1964; Néda et al., 2000; Okazaki et al., 2015; Ramirez et al., 2016; Strogatz, 2003). A study particularly relevant to our research involves the synchronization and coordination of human behavior using the Dynamical Systems Approach (Haken et al., 1985; Schmidt et al., 1990). These studies have investigated the timing matching of behaviors, such as leg swings and rocking chair movements. It was suggested that the behaviors of these individuals tend to stable in either in-phase synchronization, where actions are perfectly timed, or anti-phase synchronization, where actions occur at opposite times (Richardson et al., 2007; Schmidt et al., 1990; Schmidt and O'Brien, 1997). Furthermore, it has been proposed that in-phase synchronization is more stable and easier to transition into compared to anti-phase synchronization. These studies represent efforts to understand the mechanisms of synchronization and entrainment from the perspective of the dynamical systems approach. Defining synchronization clearly can be challenging, as different research domains use varying definitions. However, based on the definition and the features of phenomena in psychology, the current study defines “synchronization” as the periodic repetition of similar actions with matched timing, like in-phase synchronization and anti-phase synchronization (e.g., Bernieri and Rosenthal, 1991; Fujiwara and Daibo, 2016). Additionally, we define “coordination” as encompassing a broader range of behavior matching, which includes not only synchronization but also leader-follower relationships and polyrhythms (Konvalinka et al., 2010). In these forms of behavior matching, deviations in timing (leader-follower relationships) or period (polyrhythms) are observed among individuals' actions.

A similar emphasis on synchronization and coordination has been gradually gaining attention in the domain of the performing arts. For example, studies examining the hand movements of two pianists during improvisations revealed that their movements tended to align in in-phase synchronization (Walton et al., 2015, 2018). Similarly, Kimmel and Preuschi (2015) investigated the head movements of tango dance pairs and found that these movements also tended to follow in-phase synchronization. These studies primarily focused on performers' interactions within a collaborative context, where multiple performers aim to achieve a shared goal, such as delivering a captivating and well-structured performance. The findings indicated that, in this collaborative context, performers often coordinated their movements in an in-phase synchronous manner.

While the studies mentioned above have provided valuable insights, certain genres of the performing arts, such as jazz sessions and breaking battles, can exist within a different context—competition. In this competitive setting, performers actively compete with one another, striving to deliver more captivating performances than their counterparts. Several qualitative studies have suggested that, in such competitive contexts, performers engage in unique interactions. For example, Shimizu and Okada (2013) qualitatively investigated battle scene in dance and proposed that dancers sometimes reference and build upon the performances of their co-dancers. These dancers focused on specific aspects of their co-dancers' performances, occasionally incorporating and developing them within their own performances. However, only a limited number of studies have quantitatively investigated these interactions among performers in a competitive context.

Several studies examining interpersonal interactions in competitive contexts have primarily focused on everyday conversations (Abney et al., 2014; Paxton and Dale, 2013, 2017). These studies compared the coordination of participants during collaborative conversations (e.g., discussions about personal interests such as their favorite music and TV programs) with their coordination in competitive dialogues (e.g., debates on social issues). The findings indicated that, in competitive contexts, individuals exhibited more frequent time-lagged coordination (behaving similarly at different times, akin to anti-phase synchronization). Furthermore, compared to collaborative contexts, participants in competitive conversations demonstrated significantly less in-phase synchronization of their movements.

In the domain of performing arts, although the number of studies remains limited, we reference two studies that have examined performers' interactions in competitive contexts. Keller et al. (2017) explored coordination among traditional chorus singers in Germany. In their study, a situation was created where girls of the same age appreciated their singing, thereby enhancing the competitive context among the singers. The study compared the coordination of their voices in this context with other situations. The results indicated that, in the competitive context, bass singers emphasized their individual voices while maintaining harmony with other parts (Alto, Soprano, Tenor). Additionally, Shimizu and Okada (2021) examined coordination among expert breakdancers during battle scenes. This study employed relative phase analysis to investigate the coordination of rhythmic movements among the dancers. The findings revealed that the dancers dynamically adjusted their coordination patterns according to the context. When not performing, i.e., before or after the battle (when the competitive context was relatively weak), the dancers synchronized their rhythmic movements in-phase, aligning their rhythms. In contrast, during the performance (when the competitive context was more intense), the dancers exhibited anti-phase synchronization, where they moved in opposite rhythmic timings.

These previous studies suggest that in competitive contexts, performers' behaviors often exhibit anti-phase synchronization. They align certain aspects of their behavior, such as frequency, while simultaneously differentiating other aspects, such as timing. However, to fully capture the dynamics of performers' interactions in competitive settings, further investigation is needed in two key points. First, it is essential to explore coordination across multiple expressive channels. As suggested by studies on traditional choruses and breaking battles, performers often coordinate specific behaviors, such as their voices or rhythmic movements, in an anti-phase synchronous fashion. However, in real performance contexts, performers engage in active interactions through a variety of expressive channels, including facial expressions, gestures, rhythmic movements, and back-and-forth movements. In these performance situations, performers must coordinate these diverse channels, sometimes forming a complex and comprehensive state of coordination. A deeper examination of this multichannel coordination is necessary to more accurately capture the nature of performers' interactions.

Second, it is crucial to investigate why anti-phase synchronization occurs more frequently in competitive contexts. The studies mentioned above have not explored this aspect in detail. Drawing from the open communication nature inherent in the performing arts (Okamoto, 2008; Okamoto et al., 2005), Shimizu and Okada (2021) proposed that the specific situations and goals of the performing arts, which involve performers showcasing their interactions to an audience, may facilitate this anti-phase synchronization. In a competitive context, each performer aims to highlight their own performance and demonstrate superiority to the audience. Shimizu and Okada (2021) speculated that performers may contrast their performances with those of others by concurrently aligning and misaligning some aspects of their actions, in an effort to emphasize the uniqueness of their performance. This dynamic, they suggested, leads to frequent anti-phase synchronization. However, this hypothesis has not been sufficiently investigated. To deepen our understanding, it is necessary to explore the underlying factors contributing to anti-phase synchronization in competitive contexts. Additionally, a more detailed investigation into how the open-communication nature of these contexts shapes the features of performers' interactions is required.

In light of these findings, we turned our attention to another expressive modality in dance—namely, back-and-forth movements—and investigated how such movements are coordinated among expert dancers in battle performances. While Shimizu and Okada (2021) demonstrated anti-phase synchronization in dancers' rhythmic movements, their study did not explore coordination across other expressive channels. Given the role of rhythmic coordination in dance, it is reasonable to speculate that similar coordination patterns, such as anti-phase synchronization, may also emerge in other movement dimensions. To test this hypothesis, we focused specifically on the coordination of back-and-forth movements. In the context of dance performance, practitioners often emphasize the importance of spatial positioning between dancers. Moreover, psychological research on personal space suggests that back-and-forth movement and the relative distance it creates between individuals can serve as a subtle but significant factor in regulating social interactions (hidden dimension, e.g., Hall, 1966; Hayduk, 1978; Kennedy et al., 2009). These perspectives support the idea that back-and-forth movement may offer a valuable lens through which to investigate the interaction dynamics of dancers in battle scenes.

Furthermore, back-and-forth movement appears to be a meaningful variable for investigating the underlying mechanisms of anti-phase synchronization in competitive artistic interactions. In the present study, we aim to clarify the dynamics of such interactions by comparing them with the coordination patterns observed in competitive sports settings. Kijima et al. (2012) and Okumura et al. (2012) investigated interpersonal coordination in competitive sports such as Kendo (Japanese fencing) and tag-taking games—activities in which two players compete to take a tag from each other's clothing. These studies focused on the coordination of relative distance and back-and-forth movements between two players. Their findings revealed that the players coordinated their movements in an anti-phase manner: when one player moved forward to close the distance, the other moved backward to increase it. This approaching–retreating relationship was actively and frequently switched, with the average and modal switching interval being ~0.5 s. By actively coordinating their movements in this way, the two players maintained a consistent relative distance—~2.8 m—between them. These studies also demonstrated that such coordination patterns varied dynamically depending on the distance between the players. When the distance was shorter than ~2.8 m (the mode of relative distance), the players exhibited anti-phase synchronization; in contrast, when the distance exceeded 2.8 m, they shifted to in-phase synchronization. Moreover, these studies found that players generally maintained this coordination pattern throughout the match. It was typically disrupted only at the very end, when one player either struck the opponent or successfully removed the opponent's tag. This pattern of sustained coordination followed by sudden break was consistently observed in these forms of interpersonal competitive sports.

The aforementioned studies suggested that the coordination patterns observed in interpersonal sports, such as Kendo, are influenced by factors such as the goal of the activity (e.g., to hit an opponent's body or take their tag), the players' physical attributes, and the tools used (e.g., the length of the Japanese bamboo sword, the length of the player's arm, and the distance they can cover in a single step). These factors facilitate specific coordination patterns within the competitive framework of sports. However, in the context of performing arts, such as breaking battles, the goals and rules differ significantly from those of interpersonal sports, despite the shared competitive context. In most performing arts, physical contact with other performers is not a primary objective, and thus, performers typically do not focus on it to the same extent as athletes do. Instead, their goal is to create visually engaging interactions for the audience, as highlighted in studies on open communication (Okamoto et al., 2005; Okamoto, 2008). While athletes in interpersonal sports may occasionally pay close attention to the audience, their main focus is on the competition itself, not audience engagement. We hypothesize that these similarities (competitive context) and differences (goals and rules) between performing arts and interpersonal sports significantly influence the nature of interactions between performers and players. This study investigates the characteristics of performer interactions in dance, and compares these findings with those from previous research on sports coordination (Kijima et al., 2012; Okumura et al., 2012). By examining these interactions, we aim to uncover the unique features of performing arts-based coordination.

In light of the preceding discussion, the present study had two main objectives.

- First, we investigated the coordination of back-and-forth movements among expert breakdancers during battle performances, focusing on whether they exhibited anti-phase synchronization, similar to the rhythmic synchronization observed in previous studies.
- Second, we explored the similarities and differences between the coordination of back-and-forth movements in the performing arts (specifically breaking) and in interpersonal sports (such as Kendo and tag-taking games) (Kijima et al., 2012; Okumura et al., 2012). Through this comparison, we aimed to identify the unique features of performer interactions in competitive contexts.

We focused on expert breakdancers' battle scenes for several reasons. Breaking battle scenes, in which two dancers or teams face off and compete based on the quality of their performances, serve as a primary format for competitive interaction. Historically, breaking emerged as a form of alternative expression to gang fights (OHJI, 2001; Watkins, 2005). Furthermore, the authors have been conducting research on this performance context for an extended period, and previous studies have demonstrated that coordination among dancers can be measured and analyzed quantitatively (Shimizu and Okada, 2021). These features of breaking battles suggest that this setting provides an ideal environment for investigating performer interactions within a competitive context. In this study, we utilized the movement data collected from a previous experiment that focused on rhythmic movement coordination (Shimizu and Okada, 2021), analyzing it from a different perspective to explore the dynamics of back-and-forth movement coordination.

## 2 Methods

### 2.1 Participants

Seven expert breakdancers participated in this study (Experts A–G). Their mean age of the participants was 27.29 years (*SD* = 2.43), and the average amount of breaking experience was 10.86 years (*SD* = 2.54). All participants had previously won first or second place in breaking competitions in Japan. The experts were divided into two groups for the study (Group 1: Experts A, B, C, D; Group 2: Experts B, E, F, G) with each group participating in the experiment independently.

### 2.2 Procedures

In each group, four expert dancers were paired up to engage in battles in a round-robin format, resulting in six pairs per group. This structure led to a total of twelve battles. During each battle, the dancers performed three times in turn.

The experimental design followed a two-factor mixed design. The first factor was the type of pair as described later, comparing the Real pair condition, in which participants interacted directly with each other, and the Virtual pair condition, in which pairs were created artificially without real interaction. The second factor was the performance turn, comparing four conditions: before the performance, during the performance (A's turn and B's turn, representing the first and second dancers' performances, respectively), and after the performance. As described below, Analysis 1 focused solely on the first factor (pair type), while Analysis 2 incorporated both factors (pair type and performance turn).

**Figure 1:**
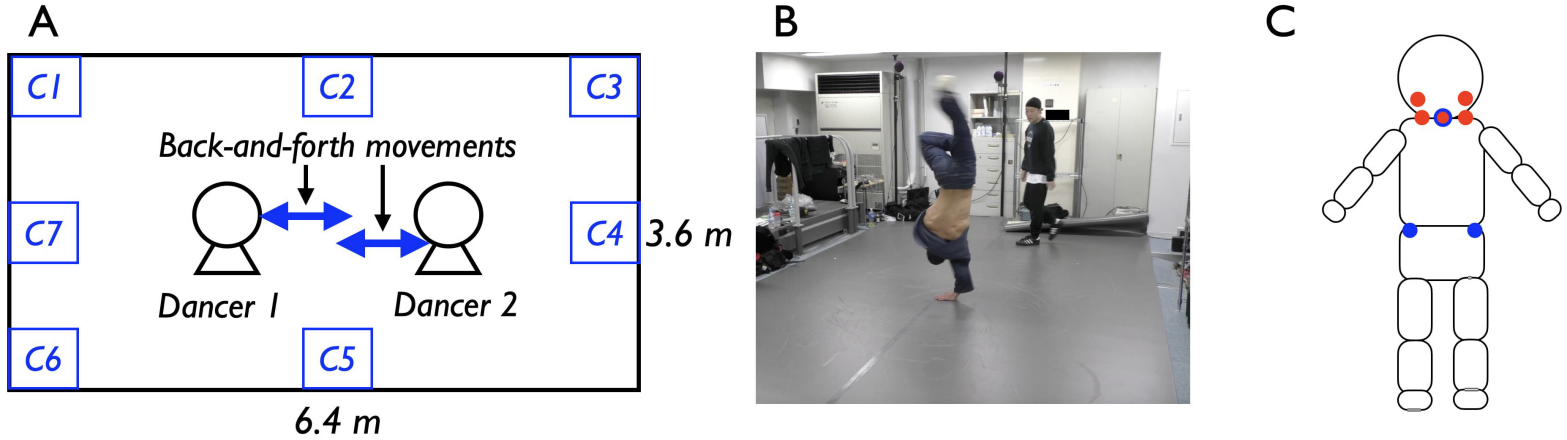
**(A)** Outline of the battle scene. Two dancers faced each other and showed their performances in turn. **(B)** Picture of the battle scene. One dancer showed his performance, and another dancer watched and responded to his performance. **(C)** Marker setting. The picture draws the back side of the participants. The red circle indicates the marker positions around the neck and blue circle indicates the marker positions around the trunks. In the analysis, we used the marker data around the neck, mainly the marker data of red circle with blue lines (for a few participants, this marker fell off during their performances, so other neck marker data was used). A portion of the figure has been adapted and modified from Shimizu and Okada (2021).

The experimental situations were designed to closely replicate real battle scenes in order to capture natural interactions between dancers (OHJI, 2001; Watkins, 2005). To achieve this, the dancers determined the performance order through their interactions during the battle. Additionally, no time limits were imposed on their performances. Based on our previous fieldwork (Shimizu and Okada, 2018), we selected a specific track, *DJ Fleg “Chelles”*, to be used across all battles; however, the dancers were not instructed to perform to any particular music. Moreover, to facilitate meaningful interactions, we consulted with the dancers in advance and ensured that the experimental space was appropriately sized for the battle (see Figures 1A, B). The space size was carefully considered, as a small space could limit the dancers' ability to interact freely. Moreover, we did not disclose the true purpose of the study to the dancers beforehand. Instead, they were informed that the study was focused on biomechanics and the individual movement features of each dancer's performance. After the experiment, the dancers were fully debriefed about the actual purpose of the study. This approach was taken to minimize the potential influence of participants' awareness of being observed, as previous studies on personal space have suggested that individuals alter their social behaviors and spatial distances when they are aware that others are observing them (Hall, 1966; Hayduk, 1978; Kennedy et al., 2009).

Each dancer's position and back-and-forth movement were measured using an infrared motion capture system (OQUS 300, QUALISYS, Göteborg, Sweden). Seven markers were placed on each dancer, based on a pilot study, at locations that would not interfere with their dance performances (five markers around the neck and two on the pelvis). Given that previous studies on personal space have most commonly relied on head position as an indicator of relative distance—since people primarily perceive distance through their eyes—we focused our analysis on the movement data from the markers placed around the dancers' necks (Figure 1C). Data from three battles were excluded from the analysis due to system malfunctions and missing markers. In breaking, where movements are often acrobatic, it was occasionally difficult to capture sufficient data for some battles due to the nature of the dancers' movements.

### 2.3 Ethics statement

The experimental procedures were conducted in accordance with the Declaration of Helsinki. Additionally, the study received approval from the Ethics Committee of the University of Tokyo. All dancers provided written informed consent, and each participant was compensated for their involvement in the study.

### 2.4 Analysis

Data preprocessing and analysis were conducted using R (version 3.5.2). Missing values in the movement data were imputed using spline interpolation, and the resulting time series were smoothed using a bandpass filter set between 1 Hz and 5 Hz. We set this frequency range based on frequency of the basic back-and-forth movements predicted by the pilot study. In addition, the application of a high-pass filter is a commonly used method to remove slow trends from the data and to facilitate accurate phase estimation of movement (de Poel et al., 2020). Following smoothing and filtering, we applied standard normalization by subtracting the mean and dividing by the standard deviation of the entire time series.

**Figure 2:**
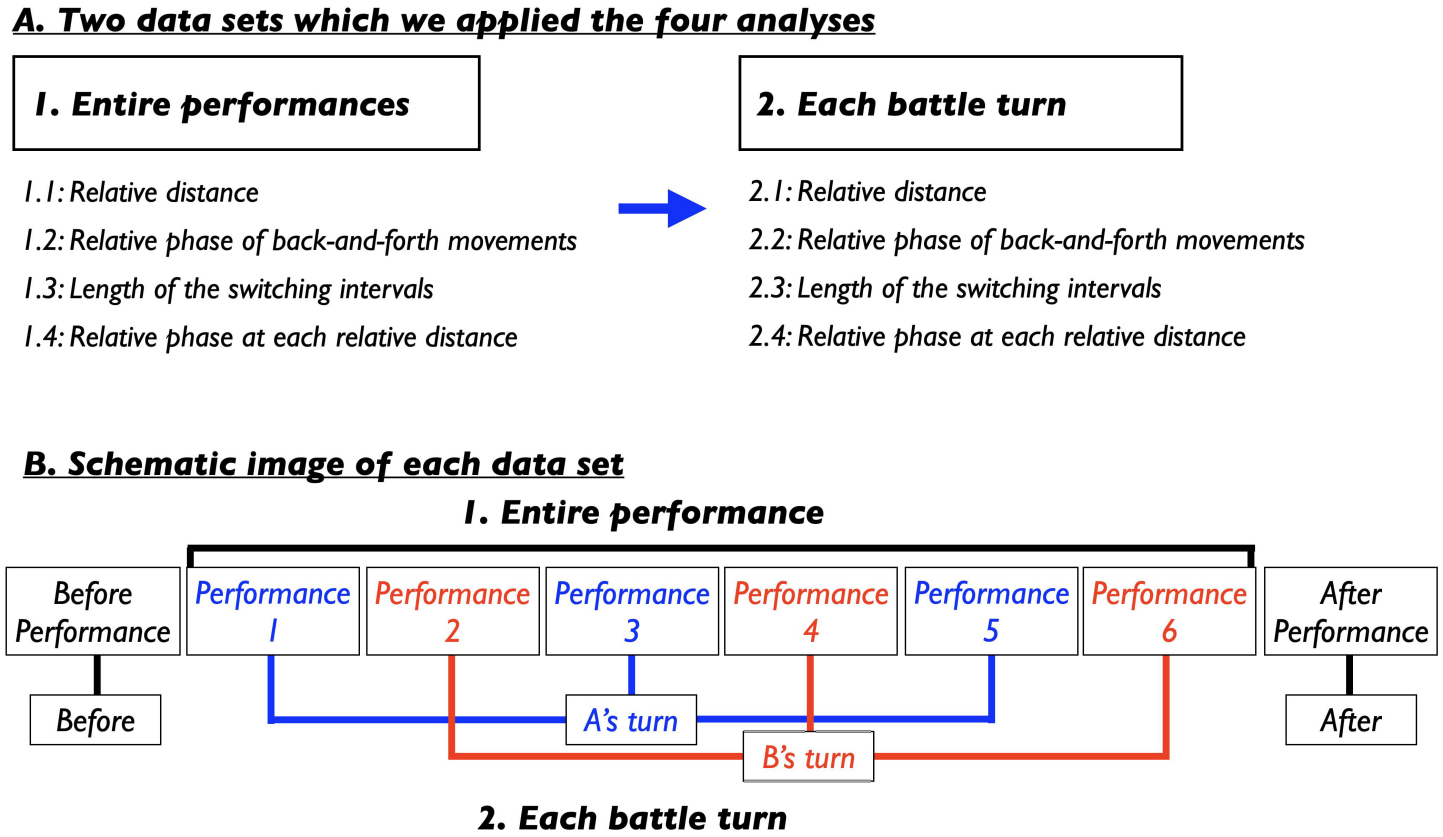
**(A)** Two data sets which we applied the four analyses. **(B)** Source images from which each dataset was extracted.

Next, we conducted four analyses on the collected data (Figure 2), each targeting a distinct aspect of the dancers' interactive movements:

1. The relative distance between the two dancers.
2. The coordination of their back-and-forth movements, quantified by the relative phase between their motions.
3. The duration of intervals at which the dancers switched movement directions, analyzed in relation to the coordination of their back-and-forth movements.
4. The coordination of back-and-forth movements as a function of relative distance between the dancers.

We conducted our analyses using a method developed in previous studies on Kendo matches and tag-taking games (Kijima et al., 2012; Okumura et al., 2012). Specifically, we applied this method to two datasets: the entire performance scenes and each individual battle turn (Figure 2). First, we examined the coordination between dancers throughout the full performance (Figure 2, top). Subsequently, we analyzed coordination within four distinct turns (Figure 2, bottom). Before performance (Before) captured the moments before the dancers began their performances; Turn of the first dancer (Performance, abbreviated in P1, P3, P5) corresponded to the performance of the first dancer; Turn of the second dancer (P2, P4, P6) to that of the second dancer; and After performance (After) to the phase after both performances had ended. For each analysis, we formulated specific hypotheses grounded in prior research on the back-and-forth and rhythmic movements observed in Kendo matches, tag-taking games, and breaking battles (Kijima et al., 2012; Okumura et al., 2012; Shimizu and Okada, 2021).

To evaluate these hypotheses, we conducted targeted multiple comparisons. This analytical strategy was adopted to account for the relatively small sample size in the current study. Moreover, had we employed exhaustive pairwise comparisons, the number of comparisons would have been excessive, potentially compromising the statistical power of the tests.

**Figure 3:**
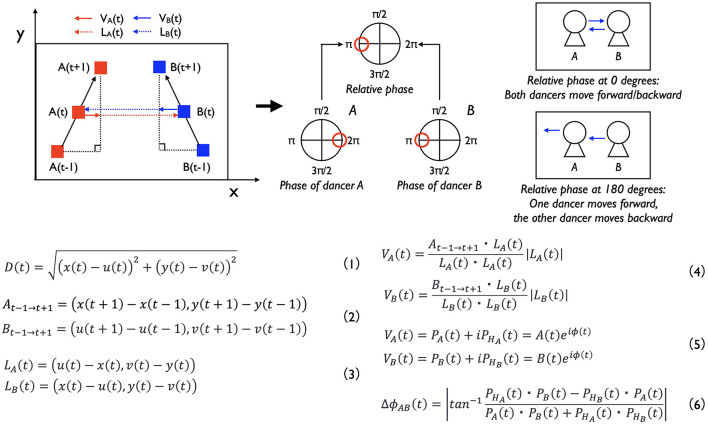
Procedures of calculating the relative distance and relative phase of the two dancers' back-and-forth movements. We made this explanation by referring to Kijima et al. (2012) and Okumura et al. (2012). *D (t)* indicates the relative distance at time *t, x (t)* and *y (t)* indicate the position of dancer A at time *t* and *u* and *v* indicate that of dancer B. *A*_*t*−1 → *t*+1_ and *B*_*t*−1 → *t*+1_ indicate the vectors of the movements from time *t-1* to time *t*+*1*. *L*_*A*_ and *L*_*B*_ show the vectors of the distance at time *t*. *V*_*A*_*(t)* and *V*_*B*_*(t)* indicate the vectors of the movements to the co-dancer's direction at time *t*. *P*_*A*_*(t)* and *P*_*HA*_*(t)* show the phase of these vectors of dancer A, and *P*_*B*_*(t)* and *P*_*HB*_*(t)* show these of dancer B. Φ_*AB*_ *(t)* show the relative phase between the two dancers at time *t*.

In the first analysis, we examined the relative distance between the two dancers throughout their performances (Figure 3). To do so, we calculated the Euclid distance between the dancers based on their movement data along the x- and y-axes (Equation 1). We then analyzed the distribution of these distances over time and identified the mode—that is, the most frequently occurring distance—within this distribution. Drawing on previous findings from studies of interpersonal coordination in sports and dance, particularly those involving anti-phase synchronization, we anticipated that the dancers would coordinate their back-and-forth movements in an anti-phase manner, thereby maintaining a consistent relative distance during their performances. Based on this expectation, we hypothesized that certain relative distances (i.e., modes) would occur significantly more frequently than others. This hypothesis was tested in the first analysis.

In the second analysis, we investigated the coordination of back-and-forth movement between the two dancers (Figure 3, Equations 2–6). This analysis involved calculating the relative phase between the dancers' movements, following procedures developed in earlier studies on Kendo matches and tag-taking games (Kijima et al., 2012; Okumura et al., 2012). First, we calculated the vector representing Dancer A's back-and-forth movement between time points *t-1* and *t*+*1* (Equation 2). We then calculated the vector representing the distance between the two dancers at time *t* (Equation 3). Next, we projected the movement vector (from *t-1* to *t*+*1*) onto the distance vector at time *t*, thereby obtaining the directional component of Dancer A's movement toward or away from Dancer B at that time point (Equation 4). This value captured the extent to which Dancer A moved forwards or backwards in Dancer B's direction at time *t*. We performed the same set of calculations for Dancer B. Subsequently, we applied the Hilbert transform to these directional movement signals to derive the instantaneous phases of the dancers' movements (Equation 5). Finally, we computed the relative phase between the two dancers' movement phases at each time point (Equation 6), which allowed us to quantify the degree and type of coordination between them.

The relative phase provides a quantitative measure of coordination between the two dancers' back-and-forth movements. A relative phase of 0 degrees indicates in-phase synchronization, meaning that both dancers moved in the same direction at the same time (e.g., when Dancer A moved forward, Dancer B also moved forward). In contrast, a relative phase of 180 degrees indicates anti-phase synchronization, where the dancers moved in opposite directions (e.g., when Dancer A moved forward, Dancer B moved backward). Although various methods exist for analyzing synchronization and coordination in human behavior, we selected this approach to allow for direct comparison with previous studies on Kendo-matches and the tag-taking games. As mentioned in the hypothesis of the first analysis, we anticipated that the dancers would exhibit anti-phase synchronization in their back-and-forth movements during their performances. Therefore, we hypothesized that anti-phase synchronization (i.e., a relative phase around 180 degrees) would occur significantly more frequently than other coordination patterns.

We also created a Virtual pair condition to serve as a baseline for comparison with the Real pair condition (e.g., Bernieri et al., 1988; Dale et al., 2011). In the Virtual Pair condition, we generated pseudo-pairs by replacing one dancer's movement data from a Real pair with that same dancer's data from a different battle. This procedure allowed us to preserve individual movement characteristics while eliminating the possibility of real-time interpersonal coordination. Previous studies have shown that behavioral synchrony can occasionally emerge by chance, even the absence of direct interaction. Therefore, by comparing the relative distance and relative phase between dancers in the Real Pair and Virtual Pair conditions, we aimed to evaluate whether the observed coordination in the Real Pairs exceeded what could be expected from chance alone.

In the third analysis, we examined the temporal characteristics of coordination by analyzing the intervals at which the dancers switched their movement directions. Specifically, we focused on the signs (positive or negative) of each dancer's movement phase at time *t*. A positive sign indicated that the dancer was moving backward, while a negative sign indicated forward movement. Following the method proposed by Kijima et al. (2012), we calculated an instantaneous product indicator by multiplying the movement phases of the two dancers at each time point. When the product was positive, both dancers were moving in the same direction (either both forward or both backward). When the product was negative, the dancers were moving in opposite directions. We then identified the time points at which this indicator changed sign from positive to negative. Such transitions signified shifts in the coordination pattern—from synchronized movement in the same direction to anti-phase movement in opposite directions. By analyzing the intervals between these transitions, we aimed to capture the temporal dynamics of coordination switching between the dancers.

We also examined which dancer moved forward at the time when coordination patterns changed, by checking the sign of each dancer's movement phase at those transition points. Using this information, we identified the exact time points at which each dancer switched their movement direction (i.e., from forward to backward, or vice versa). We then established the time when the dancers switched their moving direction (forward or backward) and calculated intervals between these time points. These intervals provided insight into whether the dancers were actively and frequently switching their movement directions, or whether they tended to maintain a single direction for extended periods. Previous study by Kijima et al. (2012) on tag-taking games found that players switched directions ~every 0.5 s on average, suggesting that quick directional changes are characteristic of real-time coordination in competitive interpersonal sports. Similar patterns have also been observed in studies on the competitive behavior of insects (Greenfield and Roizen, 1993; Greenfield et al., 2017), where rapid directional changes are thought to play a functional role in dynamic coordination. Based on these findings, we expected that dancers in battle performances would also exhibit short, regular switching intervals. Therefore, we hypothesized that specific short intervals would be observed statistically more frequently than other interval lengths.

In the fourth analysis, we examined how back-and-forth coordination varied as a function of relative distance between the dancers. Using the relative distance data calculated in the first analysis, we segmented the performance scenes according to distance ranges. For each distance segment, we calculated the frequency distribution of relative phases to identify how coordination patterns changed depending on the dancers' spatial relationship. Okumura et al. (2012), in their study on Kendo players, reported that coordination patterns shifted markedly—from anti-phase to in-phase synchronization—at a specific distance (mode: 2.8 m). However, as noted in their discussion, such distance-dependent coordination shifts may be closely tied to the task goals (e.g., striking the opponent with the bamboo sword) and the physical constraints (e.g., sword length, step length) inherent in Kendo matches. In contrast, competitive dance performances are aimed at creating visually engaging interactions for an audience, rather than achieving physical contact. Therefore, it is reasonable to speculate that dancers may not exhibit such abrupt coordination shifts at a particular distance. Instead, we expected that their coordination patterns would remain relatively stable across varying distances, without a drastic transition at a specific mode.

Furthermore, previous studies have indicated that dancers in battle performances dynamically adjust their rhythmic coordination over time and in response to contextual factors (Shimizu and Okada, 2021). These findings suggest that interpersonal coordination in such performances may be influenced more by temporal and contextual elements than by physical distance. Based on this perspective, we hypothesized that dancers would not exhibit a drastic change in coordination at any particular relative distance (i.e., mode). Instead, we expected that anti-phase synchronization would be observed more frequently than other coordination states across all distance ranges. Additionally, we hypothesized that the frequency of anti-phase synchronization would vary significantly depending on the performance context—specifically, before, during, and after the dancers' individual performances.

## 3 Results

### 3.1 Results of the entire performance

#### 3.1.1 Relative distance between two dancers

**Figure 4:**
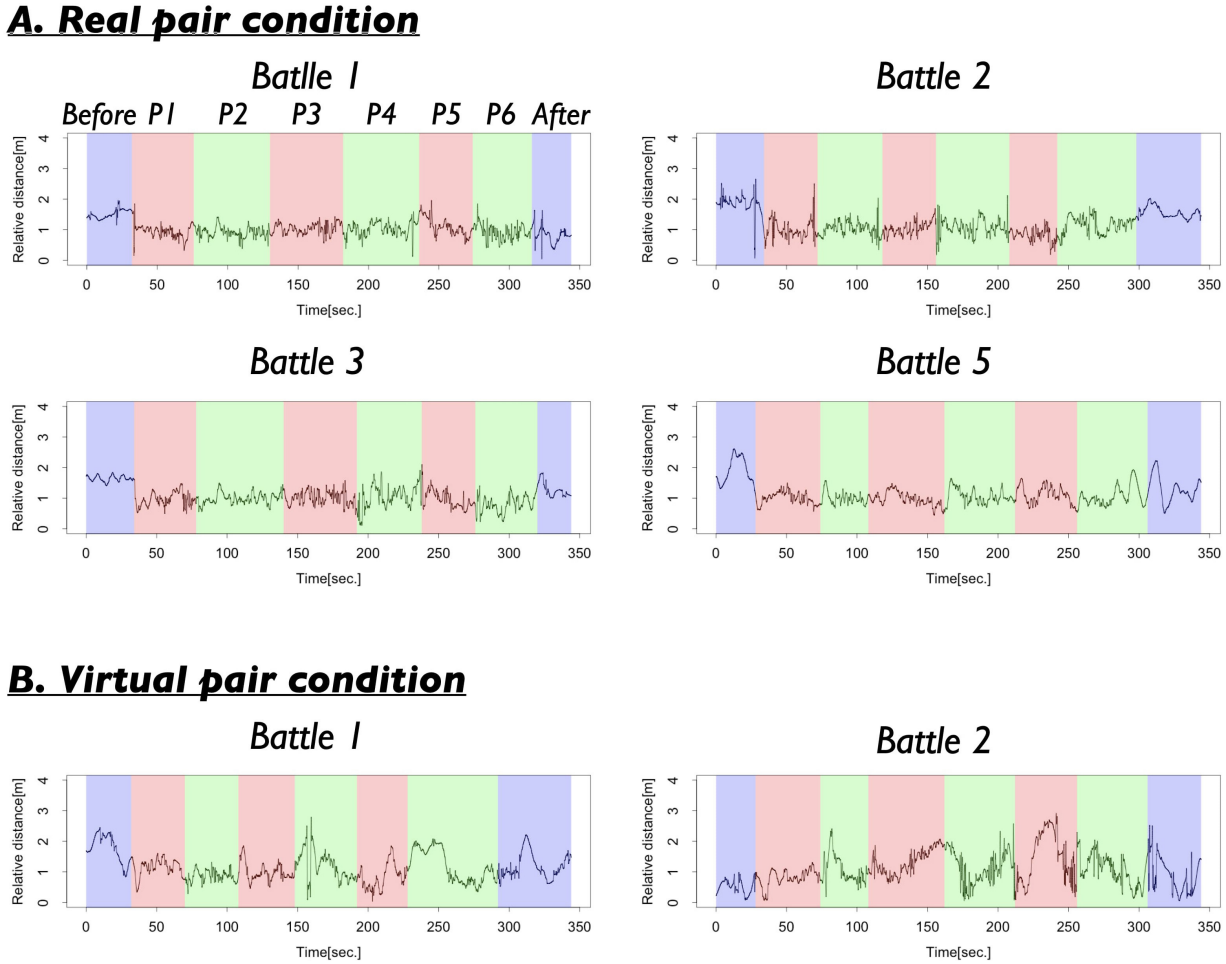
**(A)** Several examples of the relative distances in the Real pair condition. The spaces colored in blue show the time when both dancers did not show their performances (Before, After), those colored by red show the performance time of the first dancer (P1, P3, P5), and those colored by green show the performance time of the second dancer (P2, P4, P6). **(B)** Several examples of the relative distances in the Virtual pair condition.

**Figure 5:**
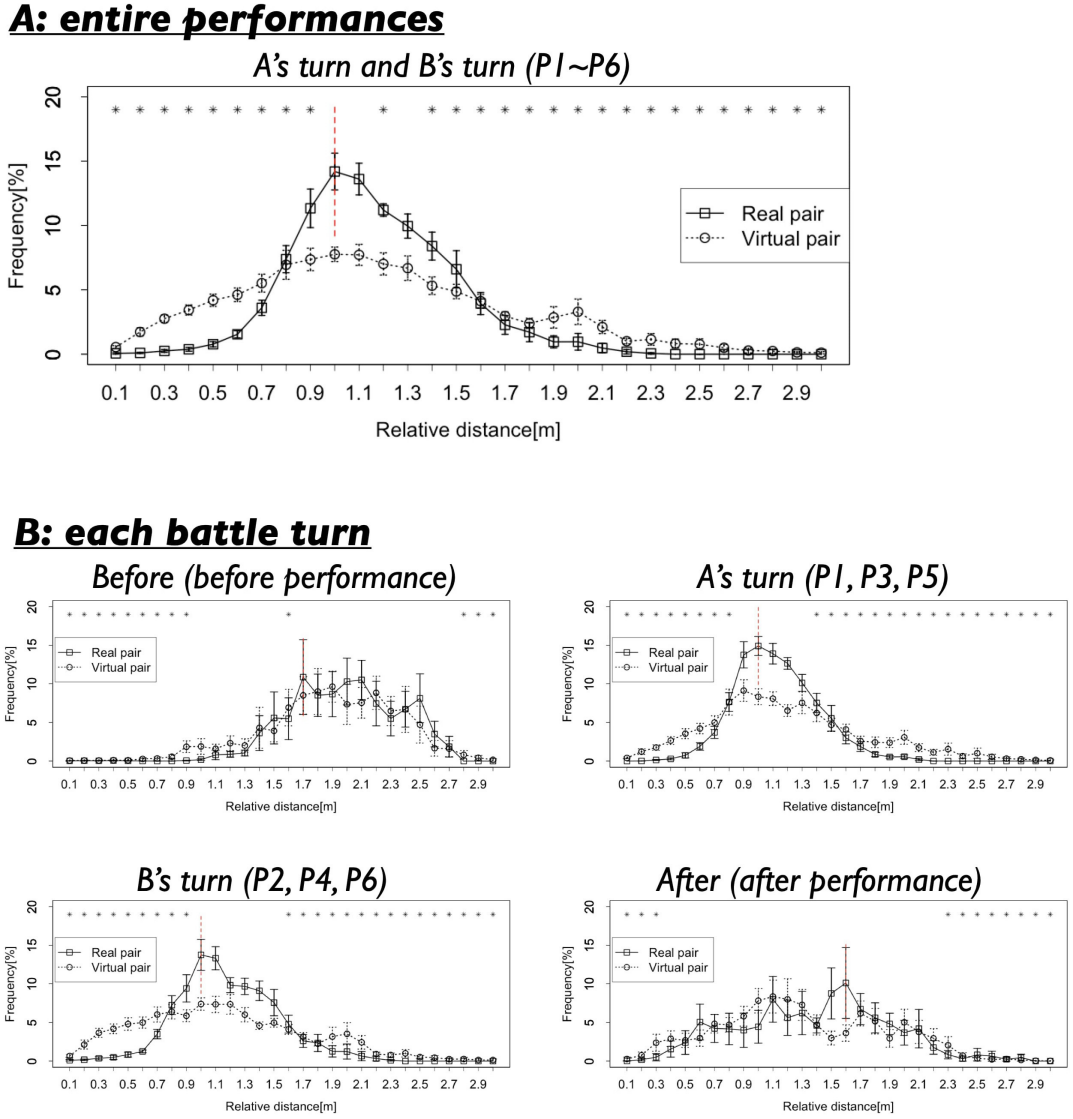
**(A)** Frequencies of the relative distances in the whole performance turns (P1–P6). Black vertical lines indicate standard error. Red vertical line indicates the mode. Asterisks indicate the relative distances whose frequencies show significant differences with the mode. *p < 0.05. **(B)** Frequencies of the relative distances in each turn.

Figures 4, 5A present the results of the first analysis, which focused on the entire performance scenes. Figure 4A illustrates that the two dancers maintained a relative distance of ~1.0 m, particularly during the performance segments (P1–P6). Figure 5A, which shows the frequency distribution of the mean relative distance for each distance category, indicates that relative distances around 0.9–1.3 m were most frequently observed in the Real pair. The distribution of relative distances for the Real pair clearly exhibited a single peak. However, Figures 4B, 5A reveal that these patterns were not observed in the Virtual pair.

The statistical test confirmed these findings. Significant differences were observed between the frequency of the 1.0 m relative distance (the mode) and most other relative distances in the Real pair (corrected using the Benjamini-Hochberg method; Benjamini and Hochberg, 1995). As indicated by the asterisk in Figure 5A, 27 out of all 29 relative distances showed significant differences (93.10%). The results were as follows: *t*_(8)_ = 0.54–19.35, *p* = 0.00000004–0.61, *d* = 0.13–8.35 (detailed results are provided in Appendix A-1). In contrast, the same comparison for the Virtual pair also revealed significant differences, but the number of relative distances that showed significant deviations from the mode (1.0 m) was more limited (24 out of 29 relative distances or 82.76%). The results for the Virtual pair were as follows: *t*_(11)_ = 0.36–15.37, *p* = 0.00000002–0.72, *d* = 0.07–7.67.

Next, we examined the differences in the mode frequencies between the Real and Virtual pairs. A comparison of the mode (both 1.0 m) revealed that the frequencies were significantly different between the Real and Virtual pair [*t*_(11.84)_ = 4.24, *p* = 0.001, *d* = 2.02]. This result suggests that the specific relative distance of 1.0 m was observed more frequently in the Real pair. During the battle scenes, the two dancers maintained this specific relative distance while performing. As shown in Figure 4A, although the relative distances were not completely fixed, they exhibited some small fluctuations.

#### 3.1.2 Relative phase of back-and-forth movements between two dancers

**Figure 6:**
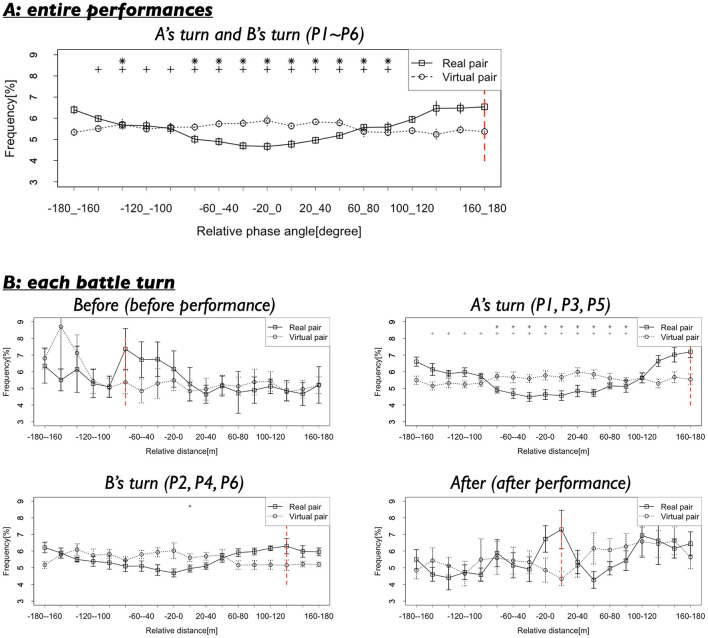
**(A)** Frequencies of the relative phases of dancers' back-and-forth movements in the whole performance turns (P1–P6). Black vertical lines indicate standard error. Red vertical line indicates the mode. Asterisks and crosses indicate the relative phases whose frequencies show significant differences with that at −180 to −160 degrees and 160–180 degrees (*p < 0.05 with −180 to −160 degrees, ^+^*p* < 0.05 with 160–180 degrees). **(B)** Frequencies of the relative phases in each turn.

Figure 6A shows the results of the second analysis. In the Real pair condition, relative phase at−180–160 and 160–180 degrees were observed with high frequency, suggesting that the two dancers exhibited anti-phase synchronization in their back-and-forth movements. In contrast, the Virtual pair did not show this pattern; rather, the frequencies across all relative phases were approximately uniform.

A statistical comparison corrected using the Benjamini-Hochberg method revealed significant differences between the frequencies of the anti-phases (−180–160 and 160–180 degrees) and most other phase ranges. As indicated by the asterisk in Figure 6A, 10 out of 17 relative phase comparisons showed significant differences for the −180–160 degrees (58.82%), and 13 out of 17 comparisons showed significant differences for the 160–180 degrees (76.47%). For the −180–160 degrees: *t*_(8)_ = 0.13–7.89, *p* = 0.002–0.90, *d* = 0.06–3.06, and for 160–180 degrees: *t*_(8)_ = 0.28–6.46, *p* = 0.002–0.83, *d* = 0.10–3.26 (see detailed results in Appendix A-2). In contrast, the same analysis for the Virtual pair did not reveal similar tendencies. The frequencies of the anti-phase ranges did not significantly differ from those of other phase ranges. For the −180–160 degrees, the results were: *t*_(11)_ = 0.06–2.20, *p* = 0.70–0.96, *d* = 0.02–0.89, and for 160–180 degrees: *t*_(11)_ = 0.05–1.73, *p* = 0.70–0.96, *d* = 0.02–0.81.

Furthermore, we compared the frequencies of anti-phase synchronization between the Real and Virtual pairs. The analysis revealed that the frequencies of the anti-phase (−180–160 and 160–180 degrees) differed significantly between the two conditions. For the −180–160 degrees: *t*_(18.39)_ = 3.97, *p* = 0.0009, *d* = 1.72, and for the 160–180 degrees: *t*_(18.42)_ = 4.24, *p* =0.0009, *d* = 1.84. These results indicate that the specific relative phases at −180–160 and 160–180 degrees were observed much more frequently in the Real pair condition. This suggests that the two dancers performed while responding sensitively to each other's movements, coordinating their back-and-forth movements in opposite directions. It is plausible that, during this coordination, the dancers maintained a consistent relative distance of ~1.0 m.

#### 3.1.3 Frequency of the length of the switching intervals of two dancers

**Figure 7:**
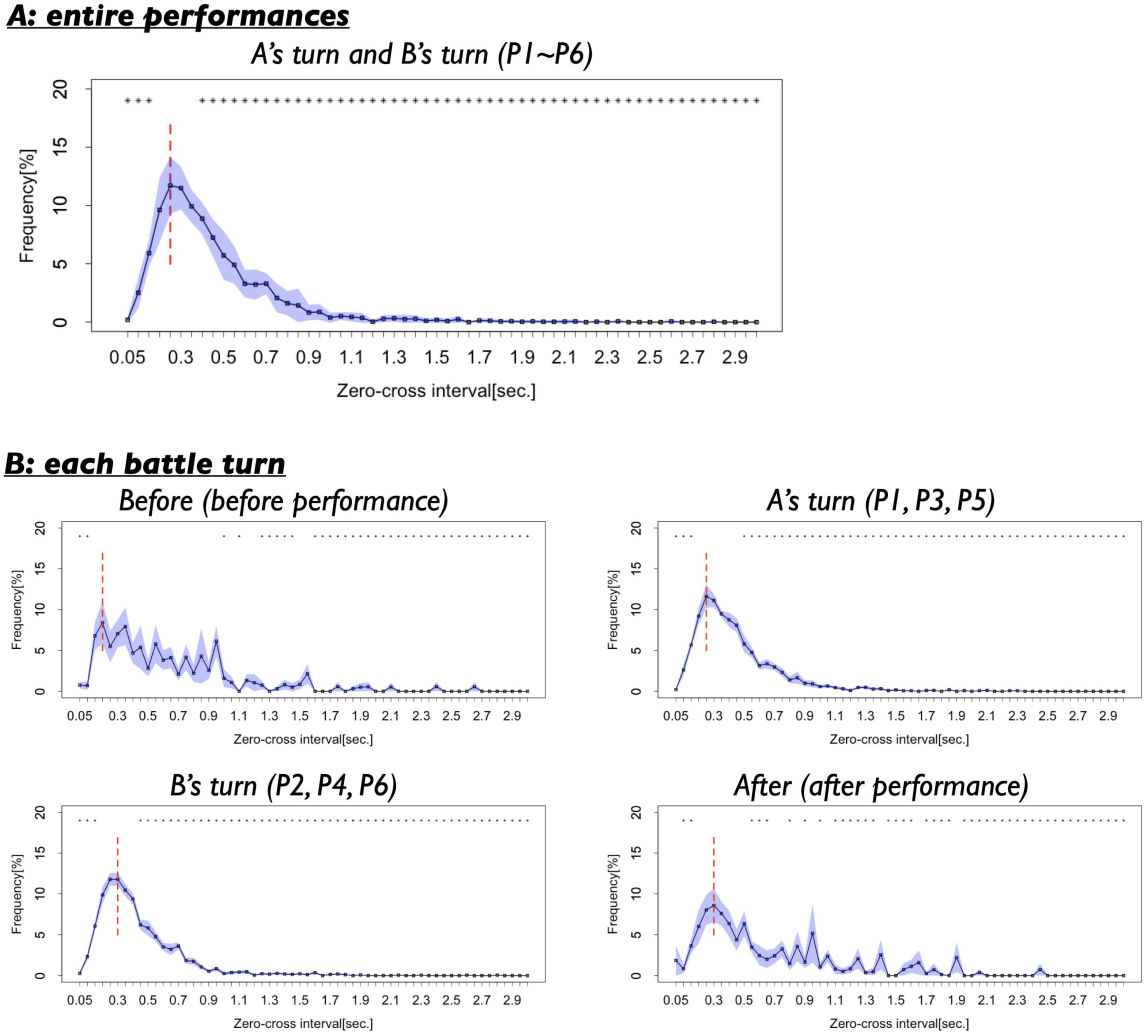
**(A)** Frequencies of the length of the switching intervals in the whole performance turns (P1–P6). Black vertical lines indicate standard error. Red vertical line indicates the mode. Asterisks indicate the length of the intervals whose frequencies show significant differences with the mode. *p < 0.05. **(B)** Frequencies of the length of the intervals in each turn.

Figure 7A presents the results of the third analysis. The figure indicates that the dancers tended to switch their forward and backward movement directions at very short intervals, ranging from 0.15 to 0.50 s, with the most frequent interval (mode) observed at 0.25 s. Statistical comparisons with Benjamini-Hochberg correction revealed significant differences between the frequency of the mode interval (0.25 s) and those of nearly all other intervals. With *t* (8) = 0.23–29.94, *p* = 0.00000001–0.82, *d* = 0.09–13.22 (see Appendix A-3 for detailed results). As indicated by the asterisks in Figure 7A, 56 out of 59 interval comparisons (94.9%) showed significant differences relative to the mode. These findings suggest that, during the battle scenes, the two dancers frequently switched their movement directions at very short time intervals. Notably, similar patterns of short-time switching of movement directions have been reported in studies examining interpersonal coordination among athletes in competitive sports settings (Kijima et al., 2012; Okumura et al., 2012). Taken together, these results highlight that active and rapid changes in movement direction are a common feature shared between the performing arts and interpersonal sports.

#### 3.1.4 Relative phase of back-and-forth movements at each relative distance

**Figure 8:**
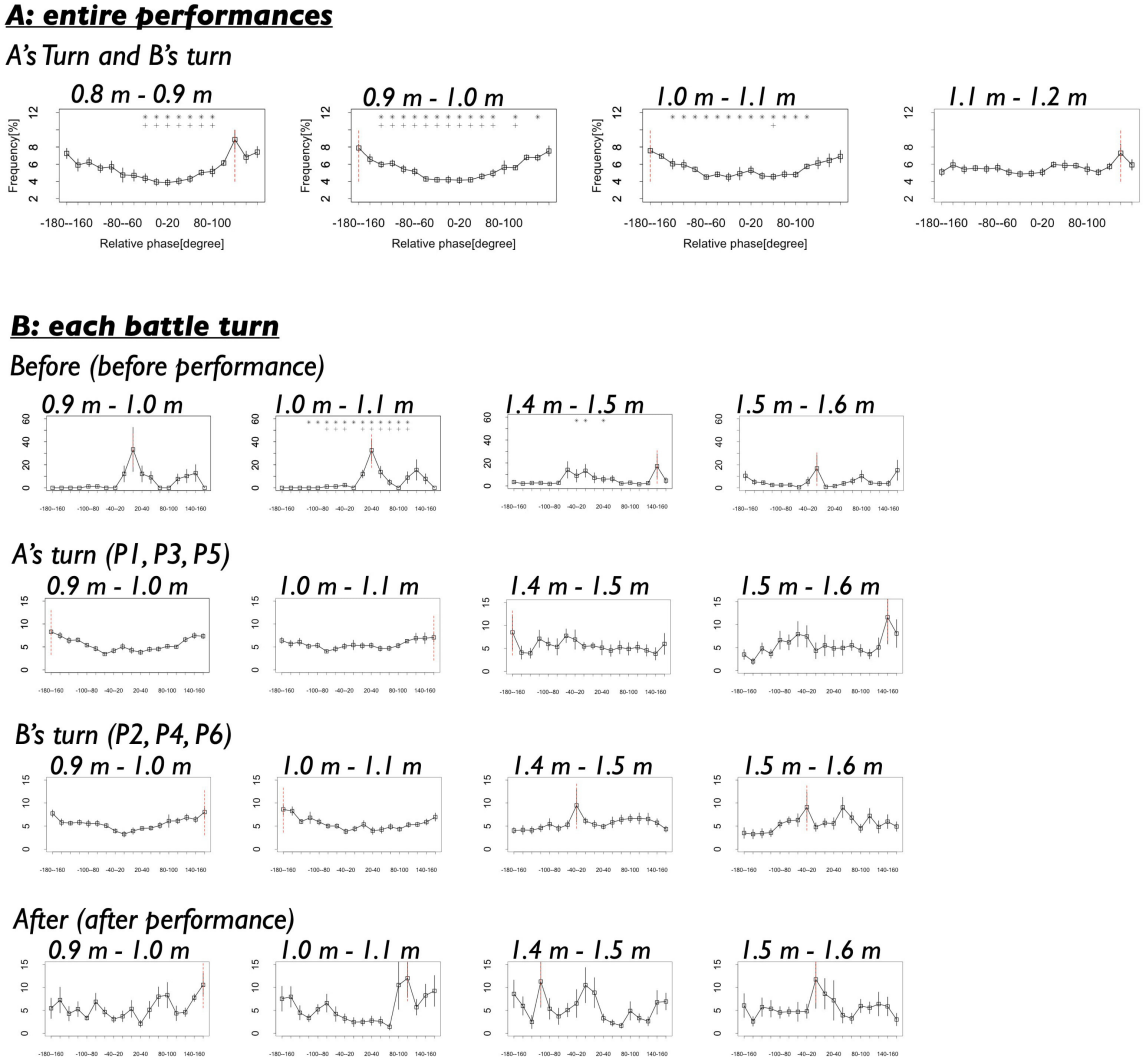
**(A)** Frequencies of the relative phases in each relative distance in the whole performance turns (P1–P6). Black vertical lines indicate standard error. Red vertical line indicates the mode. Asterisks and crosses indicate the relative phases whose frequencies show significant differences with that at −180 to −160 degrees and 160–180 degrees (*p < 0.05 with −180 to −160 degrees, ^+^*p* < 0.05 with 160–180 degrees). **(B)** Frequencies of the relative phases in each relative distance in each turn.

Figure 8A presents the results of the fourth analysis. In this analysis, we focused on relative distances around the mode value (1.0 m) observed in the Real pair condition and examined the distribution of relative phase at four distance ranges: 0.8–0.9 m, 0.9–1.0 m, 1.0–1.1 m, and 1.1–1.2 m. Figure 8A shows the results. It demonstrates that the relative phases at −180–160 and 160–180 degrees were frequently observed across nearly all distance ranges. This indicates that the dancers consistently exhibited anti-phase synchronization during their coordination, regardless of slight variations in their relative distance.

Statistical comparison with Benjamini–Hochberg correction showed that, at each distance range, the relative phase frequencies at −180—160 and 160–180 degrees were significantly different from those of most other phases, as indicated by the asterisk in Figure 8A (see Appendix A-4 for detailed results). For the 0.8–0.9 m range, −180—160 degrees: *t*_(8)_ = 0.20–6.21, *p* = 0.004–0.85, *d* = 0.09–2.13 (7 out of all 17 relative phases showed significant differences), 160–180 degrees: *t*_(8)_ = 0.20–6.36, *p* =0.004–0.85, *d* = 0.09–2.19 (7 out of all 17 relative phases showed significant differences). For the 0.9–1.0 m range, −180—160 degrees: *t*_(8)_ = 0.80–7.82, *p* =0.002–0.45, *d* = 0.25–3.46 (13 out of all 17 relative phases showed significant differences), 160–180 degrees: *t*_(8)_ = 0.80–5.54, *p* =0.005–0.45, *d* = 0.25–2.51 (12 out of all 17 relative phases showed significant differences). For the 1.0–1.1 m range, −180—160 degrees: *t*_(8)_ = 1.02–6.41, *p* = 0.005–0.37, *d* = 0.47–3.48 (13 out of all 17 relative phases showed significant differences), 160–180 degrees: *t*_(8)_ = 0.21–3.10, *p* = 0.004–0.84, *d* = 0.10–1.54 (1 out of all 17 relative phases showed significant differences). For the 1.1–1.2 m range, −180—160 degrees: *t*_(8)_ = 0.02–2.31, *p* = 0.94–0.997, *d* = 0.01–0.95 (0 out of all 17 relative phases showed significant differences), 160–180 degrees: *t*_(8)_ = 0.004–1.54, *p* = 0.94–0.997, *d* = 0.002–0.65 (0 out of all 17 relative phases showed significant differences). These results suggest that anti-phase synchronization was most pronounced when the dancers maintained a relative distance within ~0.8 to 1.1 m.

However, comparisons of anti-phase synchronization frequencies across the four distances ranges generally did not reveal significant differences. For the −180–160 degrees: *t*_(8)_ = 0.45–4.36, *p* =0.01–0.66, *d* = 0.22–2.10. For the 160–180 degrees: *t*_(8)_ = 0.13–2.12, *p* =0.40–0.90, *d* = 0.07–0.89. Significant differences were found only between the 1.1–1.2 m range and other distance ranges for the −180–160 degrees (see detailed results in Appendix A-4). These findings suggest that the dancers maintained anti-phase synchronization consistently across all distances around the mode, without dynamic changes based on their relative distance. This pattern contrasts with findings from studies on interpersonal sports, where coordination between two players shifts dramatically between anti-phase and in-phase synchronization depending on critical distances (e.g., proximity to reach or attack range, Kijima et al.,). Thus, we speculate that, during the breaking battle scenes, the dancers did not adjust their movement coordination based on physical factors such as arm length or the typical distance covered by a single step.

### 3.2 Results of each turn

#### 3.2.1 Relative distance between two dancers

Next, we examined the results across four different turns. Before performance (Before) corresponded to the period before the dancers began their performances. A's turn represented periods during which the first dancer performed, and B's turn represented periods during which the second dancer performed. After performance (After)corresponded to the period after both dancers had completed their performances. Figure 5B shows the distribution of relative distances for each turn. In the Real pair condition, relative distance around 1.0 m were frequently observed during A's turn and B's turn, when the dancers were actively performing. The mode of the relative distances for these turns was 1.0 m. In contrast, during Before, relative distances were clustered around 1.7 m (mode = 1.7 m), and during After, a similar pattern was observed, with the mode at 1.6 m. In the Virtual pair condition, however, the distribution of relative distances remained relatively stable across A's turn, B's turn, and After, without notable shifts. Furthermore, across all turns, the frequency of the modal relative distances in the Virtual pair was lower compared to that in the Real pair, suggesting less pronounced coordination in spatial positioning.

The statistical test supported these observations. Comparison corrected using the BH correction confirmed that, in each turn, the frequency of the modal distance was significantly different from the frequencies of many other distances, as indicated by the asterisk in Figure 5B. In particular, during A's turn and B's turn, when the dancers were actively performing, the mode frequency showed significant differences from nearly all other distance ranges. Specifically, Before: 13 out of all 29 distances showed significant differences from the mode (1.7 m, 44.83%), *t*_(8)_ = 0.02~3.11, *p* = 0.04–0.99, *d* = 0.008–1.47. A's turn: 25 out of all 29 showed significant differences from the mode (1.0 m, 86.21%), *t*_(8)_ = 0.69–25.98, *p* = 0.000000004–0.51, *d* = 0.27–10.58. B's turn 3: 24 out of all 29 showed significant differences from the mode (1.0 m, 82.76%), *t*_(8)_ = 0.18–12.08, *p* = 0.000007–0.86, *d* = 0.04–5.68. After: 11 out of all 29 showed significant differences from the mode (1.6 m, 37.93%), *t*_(8)_ = 0.15–4.30, *p* = 0.03–0.89, *d* = 0.05–2.03 (detailed results are provided in Appendix B-1). These findings suggest that during active performance phases (A's turns and B's turn), the dancers maintained a consistently close relative distance (around 1.0 m), whereas before and after the performances (Before and After), their relative positioning was more variable.

Further, we examined the differences in frequency of specific relative distances across turns. Comparisons with BH correction revealed that the frequency of 1.0 m—the modal distance during A's turn and B's turn— showed significant differences between several pairs of turns (Specifically, Before–A's turn, Before–B's turn, A's turn–After, B's turn–After). The statistics were *t*_(8)_ = 0.83–17.23, *p* = 0.0000004–0.43, *d* = 0.30–7.98. In contrast, the frequencies of 1.6 m (the mode in After) and 1.7 m (the mode in Before) did not show significant differences across turns. For 1.6 m: *t*_(8)_ = 1.02–1.94, *p* =0.26–0.34, *d* = 0.49–0.81. For 1.7 m: *t*_(8)_ = 1.06–1.57, *p* = 0.48–0.88, *d* = 0.09–0.74. These results suggest that the modal relative distance varied across the four turns. Before their performances (Before), dancers tended to maintain a distance of around 1.7 m. During their performances (A's turn and B's turn), they moved significantly closer, maintaining distances around 1.0 m. After their performances (After), they maintained distances of around 1.6 m. However, the tendency to maintain a specific distance was relatively weaker before or after the performances compared to during the performances. In sum, these findings indicate that the dancers actively adjusted their relative distances depending on the context of the battle scene.

#### 3.2.2 Relative phase of back-and-forth movements between two dancers

We further examined the results of the second analysis across the four turns, as presented in Figure 6B. This figure shows that, during A's turn and B's turn—when the dancers were actively performing—the relative phases at −180—160 and 160–180 degrees were frequently observed in the Real pair condition. This pattern indicates that the dancers moved forward and backward in an anti-phase synchronization. In contrast, during Before and After—before and after the performances—this tendency was not observed. Before the performances (Before), relative phase around −80—60 degrees were more frequently observed, rather than the at −180—160 and 160–180 degrees. After the performances (After), relative phase around 0–20 degrees became predominant. These findings suggest that the dancers dynamically modulated their coordination patterns depending on the context. Specifically, their coordination shifted from a leader-follower relationships (with the first dancer acting as the leader and the second as the follower) before the performance, to anti-phase synchronization during the performance, and then to in-phase synchronization after the performance.

Statistical comparison with BH correction partially supported these findings. The comparisons confirmed that, particularly during A's turn—and to a lesser extent during B's turn—the relative phase frequencies at −180—160 and 160–180 degrees were significantly different from those of most other phases, as indicated by the asterisk and cross in Figure 6B. For A's turn, −180–160 degrees: 10 out of all other 17 phases showed significant differences (58.82%), *t* (8) = 0.07–4.74, *p* = 0.005–0.94, *d* = 0.03–2.59. For 160–180 degrees: 14 out of all other 17 phases showed significant differences (82.35%), *t*_(8)_ = 0.45–7.73, *p* = 0.002–0.69, *d* = 0.16–2.93. For B's turn, −180—160 degrees: 1 out of all other 17 phases showed significant differences (5.88%), *t*_(8)_ = 0.05–4.91, *p* =0.04–0.97, *d* = 0.02–1.78. For 160–180 degrees: 0 out of all other 17 phases showed significant differences (0.00%), *t*_(8)_ = 0.03–3.70, *p* = 0.08–0.97, *d* = 0.01–1.76. In contrast, during Before and After, the relative phase frequencies at −180—160 and 160–180 degrees did not show significant differences compared to other phases (details results are presented in Appendix B-2).

Additionally, before the performances (Before), the frequency of the modal relative phase (−80–60 degrees) was not significantly different from the frequencies of other phases, although the effect sizes were relatively large. −80–60 degrees: 0 out of all other 17 phases showed significant differences (0.00%), *t*_(8)_ = 0.36–2.58, *p* = 0.33–0.73, *d* = 0.19–1.01. In contrast, after the performances (After), the frequency of the modal relative phase (0–20 degrees) was significantly different from those of other phases. 0–20 degrees: 0 out of all other 17 phases showed significant differences (0.18%), *t*_(8)_ = 0.30–4.91, *p* = 0.02–0.78, *d* = 0.006–0.70. These results suggest that the dancers dynamically adapted their movements' coordination across different turns. However, the leader-follower relationship hypothesized for Before was not strongly supported by statistically significant differences, despite the presence of moderate to large effect sizes.

Further, we examined the frequency differences of key relative phases across turns. Comparisons with BH correction revealed that the frequency of the relative phases at −180—160 and 160–180 degrees were not significantly different across turns, although the effect sizes were relatively large. −180—160 degrees: *t*_(8)_ = 0.15–1.63, *p* = 0.68–0.89, *d* = 0.06–0.84. 160–180 degrees: *t*_(8)_ = 0.48–2.88, *p* = 0.12–0.64, *d* = 0.24–1.40. Similarly, the frequencies of the modal relative phases Before and After did not differ significantly across turns, although again the effect sizes were relatively large. For −80–60 degrees: *t*_(8)_ = 0.49–1.86, *p* = 0.39–0.64, *d* = 0.23–0.94. For 0–20 degrees: *t*_(8)_ = 0.65–2.85, *p* =0.13–0.53, *d* = 0.31–1.38 (see detailed results in Appendix B-2).

Based on these results, we can speculate that the dancers actively adapted their coordination of back-and-forth movements according to the evolving dynamics of the battle situation. In particular, during their performances, the dancers tended to sustain an anti-phase synchronization, moving in opposite directions. Taking into account the findings from the first analysis as well, we propose the following sequence: Before their performances, the dancers maintained a relative distance of ~1.7 m by moving forward and backward in the same direction, albeit with some time lags between them. During their performances, they adjusted to a closer distance of around 1.0 m, reacting sensitively to each other's movements and moving in opposite directions (anti-phase coordination). After completing their performances, they returned to maintaining a relative distance of ~1.6 m again moving in the same direction.

#### 3.2.3 Length of the switching intervals of two dancers

Figure 7B presents the results of the third analysis across the four turns. The figure suggests that before and after the performances (Before and After), the intervals between direction switches varied widely, ranging from 0.15 to 1.00 s. In contrast, during the performances (A's turns and B's turn), short intervals of ~0.25 and 0.30 s were frequently observed, with the distribution exhibiting a clear single peak. There findings indicate that interval variability was relatively high during Before and After, whereas during in A's turn and B's turn, the dancers maintained more consistent, shorter switching intervals, reflecting tighter temporal coordination while performing.

Comparisons with BH correction showed that, across all turns, the frequency of the modal intervals was significantly different from those of many other intervals, as indicated by the asterisk in Figure 7B. Turn 1: 38 out of all 59 distances showed significant differences with the mode (0.20 s., 64.40%), *t*_(8)_ = 0.04–3.81, *p* = 0.01–0.97, *d* = 0.02–1.76. Turn 2: 54 out of all 59 distances showed significant differences with the mode (0.25 s., 91.53%), *t*_(8)_ = 0.17–14.81, *p* = 0.000001–0.87, *d* = 0.07–6.73. Turn 3: 55 out of all 59 distances showed significant differences with the mode (0.30 s., 93.22%), *t*_(8)_ = 0.02–29.57, *p* = 0.000000007–0.99, *d* = 0.006–13.52. Turn 4: 44 out of all 59 distances showed significant differences with the mode (0.30 s., 74.58%), *t*_(8)_ = 0.007–4.87, *p* = 0.003–0.99, *d* = 0.002–2.30 (detailed results are provided in Appendix B-3).

We also examined differences in interval frequency among turns, focusing particularly on the frequencies of the modal intervals for each turn (Before: 0.20 s., A's turn: 0.25 s, B's turn: 0.30 s, and After: 0.30 s). Comparisons with BH correction revealed that the mode frequencies in A's turn (0.25 s) and B's turn (0.30 s) were significantly different from that in Before. For 0.25 s: *t* (8) = 0.25–2.68, *p* = 0.003–0.81, *d* = 0.12–1.27. For 0.30 s: *t*_(8)_ = 0.34–2.46, *p* = 0.04–0.74, *d* = 0.17–1.10. However, the frequency of the 0.20 s interval (Before) was not significantly different across turns. *t*_(8)_ = 0.42–1.20, *p* = 0.27–0.69, *d* = 0.16–0.55 (details results are presented in Appendix B-3).

These results suggest that the dancers frequently switched their forward and backward movement directions at very short intervals, particularly during their performances. In contrast, before and after performing, the switching intervals were relatively longer and exhibited greater variability. This context-dependent modulation of switching intervals highlights a flexible adaptation of movement coordination to performance demands—a phenomenon that has not been sufficiently explored in studies of interpersonal interactions among sports players.

#### 3.2.4 Relative phase of back-and-forth movements at each relative distance

Lastly, we examined the results of the fourth analysis across the four turns, as presented in Figure 8B. In this analysis, we focused on relative distances around the modal values for each turn (1.0 m and 1.6 m) and investigated the distribution of relative phase within specific distance ranges (0.9–1.0 m, 1.0–1.1 m, 1.4–1.5 m, and 1.5–1.6 m). Figure 8B shows that before the dancers began their performances (Before), relative phases indicative of in-phase synchronization (-20–0 and 0–20 degrees) were frequently observed across all distances. In contrast, during the performances (A's turn and B's turn), relative phases corresponding to anti-phase synchronization (-180–160 and 160–180 degrees) were predominantly observed across almost all distance ranges. After the performances (After), a mixed pattern emerged: anti-phase synchronization (-180–160 and 160–180 degrees) was frequently observed at shorter distances (0.9–1.0 m and 1.0–1.1 m) while in-phase synchronization (-20–0 and 0–20 degrees) was more common at larger distances (1.4–1.5 m and 1.5–1.6 m). These results suggest that the dancers' coordination patterns were primarily context-dependent—shaped by the performance phase—rather than strictly dependent on inter-dancer distance, except during after the performances (After) where some distance-dependent tendencies were observed.

The statistical test partly supported these findings. Comparisons with BH correction revealed that, at each distance, the relative phase frequencies associated with anti-phase synchronization differed across turns. For the distance ranges at 9.0–1.0 m and 1.0–1.1 m, the frequencies at −180—160 and 160–180 degrees were significantly different among turns (detailed results are provided in Appendix B-4).

Further, we compared the frequencies of anti-phase synchronization across distances within each turn, applying BH correction. The results showed that significant differences among distances were observed only in A's turn and B's turn at 160–180 degrees (detailed results are provided in Appendix B-4).

Additionally, we compared the frequencies of anti-phase synchronization (−180—160 and 160–180 degrees) with those of other relative phases at each distance within each turn (it should be noted that we were unable to statistically test some frequency differences in Before because the dancers rarely exhibited multiple distinct relative phases during that turn, resulting in insufficient data). The results indicated that before the performances (Before), although formal statistical test was limited, the observed frequency differences between anti-phase synchronization and other relative phases were either significant or associated with large effect sizes. In A's turn, the frequencies at −180—160 and 160–180 degrees were significantly different from those of other phases across distances. In B's turn, anti-phase synchronization was frequently observed at 1.0–1.1 m distance range, but was rare at 1.4–1.5 m and 1.5–1.6 m. After the performances (After), anti-phase synchronization was frequently observed at 1.0–1.1 m and 1.4–1.5 m, but was seldom observed at 1.5–1.6 m (detailed results are provided in Appendix B-4).

These results align closely with the patterns observed in the figure. They suggest that before and during the dancers' performances, coordination patterns did not exhibit the distance-dependent characteristics that became evident after the performances. Specifically, the dancers' coordination—including the emergence of in-phase and anti-phase synchronization—did not show rapid, distance-dependent transitions. Instead, the coordination patterns were strongly shaped by the broader battle context. This context-dependence differs markedly from the coordination patterns typically observed among sports players, where rapid distance-dependent changes often occur. These findings highlight a unique and intriguing feature of performers' interactions during competitive artistic performances.

## 4 Discussion

This study investigated the interactions between expert breakdancers during battle scenes as a representative form of competitive performance. Specifically, we examined the coordination of the dancers' back-and-forth movements using relative phase analysis. The results revealed that throughout the battle scenes, the dancers frequently coordinated their movements in an anti-phase synchronization pattern—when one dancer moved forward, the other moved backward. They actively switched their movement directions at short intervals and maintained a consistent relative distance of ~1.0 m. Unlike interpersonal sports players (Okumura et al., 2012), the dancers did not exhibit drastic changes in their coordination patterns based on relative distances. Furthermore, when the battle scenes were divided into four distinct turns, it became evident that the dancers dynamically modulated their coordination patterns depending on the performance context and the timing within the interaction.

Specifically, before the performance, when the competitive context was relatively weak, the dancers frequently moved forward and backward in the same direction, albeit with some time lags, and maintained relatively long distances between each other. During the performance, when the competitive context intensified, they frequently moved in opposite directions (exhibiting anti-phase synchronization) and maintained short distances, placing them in close proximity. After the performance, the dancers again tended to move in the same direction (in-phase synchronization) while maintaining longer distances.

Based on the results of this study and previous research (Shimizu and Okada, 2021), we confirmed that performers exhibit consistent coordination patterns, such as anti-phase synchronization across several expressive channels in competitive contexts. Previous studies investigating breaking battle scenes have suggested that dancers coordinate their rhythmic movements in an anti-phase synchronization pattern (Shimizu and Okada, 2021). However, these earlier studies did not examine whether similar coordination patterns emerge across other different expressive channels under the same competitive conditions, nor did they explore the similarities and differences in coordination across multiple channels. As discussed in the introduction, performers are expected to interact through multiple expressive channels simultaneously. To fully capture the complexity of performer interactions, it is essential to consider the overall coordination state across these channels. The present study demonstrated that performers exhibited similar coordination patterns—specifically, anti-phase synchronization—across multiple expressive channels during strong competitive contexts, thereby offering an important first step toward a comprehensive scientific understanding of performer interactions. Future research should continue to develop methods and analyses capable of capturing the correspondence of coordination patterns across multiple expressive channels.

This study further illuminated important details and underlying aspects of performers' interactions. In addition to demonstrating anti-phase synchronization in the dancers' movements, we found that the dancers actively switched their forward and backward movement directions at short intervals. They also dynamically adjusted their coordination patterns depending on the performance context (i.e., performance turns). Notably, these coordination patterns were not influenced by changes in relative distance. We speculate that this context-dependent modulation of coordination is a fundamental characteristic of performer interactions. In contrast, similar coordination patterns were not observed in studies of interpersonal sports interactions, such as Kendo matches and tag-taking games (Kijima et al., 2012; Okumura et al., 2012). Specifically, in interpersonal sports, players' coordination was strongly dependent on relative distance rather than on contextual changes. Thus, the present findings highlight a key distinction between artistic performance and sports interaction: performers exhibit flexible, context-driven coordination dynamics, whereas sports players rely more heavily on distance-dependent coordination.

Careful discussion is needed to understand the background of these differences. Research on interpersonal sports, such as Kendo matches and tag-taking games, has suggested that specific goals and rules (e.g., striking the opponent's body or capturing a tag attached to the opponent) as well as physical properties (e.g., the length of a bamboo sword, arm length, and the distance covered in a single step) strongly shape coordination patterns. In these sports contexts, players consistently maintained specific interpersonal distances and adapted their coordination patterns based on these distances. Furthermore, coordination patterns in sports were not notably dependent on the progression of time during the match; players maintained stable distance-dependent coordination until the match concluded.

Unlike players in interpersonal sports, performers do not aim to strike or physically contact other performers. If the primary goal were simply to avoid contact, performers would not need to maintain close distances around 1.0 m through anti-phase synchronization, especially given that the available space would have allowed them to separate much further. Instead, goals such as open communication and expressive interaction—distinct from the objectives of interpersonal sports—likely facilitated the observed coordination patterns. In the performing arts, performers aim to present attractive performances and engaging interactions for the audience. We speculate that during competitive performances, such as breaking battles, performers sought to emphasize their individual expressions by contrasting them with those of their counterparts through anti-phase synchronization. Moreover, they may have actively modulated their coordination patterns to capture and sustain the audience's attention and interest. Given their extensive experience, expert performers may have implicitly understood how dynamic coordination patterns contribute to audience engagement, thereby exhibiting such context-sensitive behaviors naturally. However, to confirm the underlying mechanisms of this coordination, further research is needed. Specifically, studies comparing coordination patterns between experts and novice performers, as well as investigation into how different coordination dynamics influence audience attention and interest, are essential.

Furthermore, we situate our study within the framework of Beyond Synchrony, which has been proposed over the past several years to better capture the complexity of human interactions in daily life. This framework seeks to extend traditional theories of synchronization and coordination by accounting for more dynamic and multi-faceted patterns of interaction (Dale et al., 2013; Wallot et al., 2016). For example, in joint action tasks where individuals aim to achieve a shared goal, participants often display different but complementary behaviors that contribute to successful goal attainment (Richardson et al., 2015). Additionally, in everyday conversations, people coordinate their behaviors across multiple channels—such as speech, facial expressions, and gestures—forming a richly interconnected system of communication (Louwerse et al., 2012). Research investigating the influence of context on conversational coordination has also been developed within this framework (Abney et al., 2014; Paxton and Dale, 2013, 2017), highlighting how interaction patterns flexibly adapt to changing communicative demands.

Our study aligns well with the Beyond Synchrony framework and can be understood as an initial attempt to focus primarily on the influence of context and multi-channel behaviors, applying this theoretical perspective to interactions among performers. However, the present study could not fully address these aspects. Specifically, we did not quantitatively investigate complementary coordination among performers, nor did we examine the correspondence across multiple expressive channels—such as facial expressions, gestures, rhythmic movements, and back-and-forth locomotion. Future research is needed to more comprehensively capture complementary coordination patterns, such as polyrhythms, and the broader state of multi-channel coordination. This will require further development of analytical frameworks that extend current methods for analyzing synchronization and coordination in complex, real-world interactions.

There are several important caveats, as also noted in Shimizu and Okada (2021). First, the number of dancers in the study was strictly limited. Due to the busy schedules of expert dancers, it was extremely difficult to recruit a larger sample and conduct group experiments. Given these constraints, we interpreted the results by carefully considering both effect sizes and significance tests. However, to more broadly generalize these findings of this study, it is essential to conduct future investigations involving a larger number of expert performers. Second, it is necessary to establish experimental conditions that more strongly reflect the contexts of coordination and competition. In the present study, we examined the influence of context by comparing different battle turns—before, during, and after performances. Nevertheless, to more directly and clearly capture the influence of context, it would be beneficial to compare conditions that explicitly differentiate competitive and cooperative settings. For instance, comparing interactions within a crew (cooperative) vs. between crews (competitive) in crew battles would offer valuable insights. In sum, the study of performers' interactions in competitive contexts is a highly promising research area that warrants further scientific exploration.

## Data Availability

The data analyzed in this study is subject to the following licenses/restrictions: this study experimented with the performances of highly accomplished dancers. Due to the nature of these dancers' work, certain restrictions were imposed on directly sharing data on the dancers' performances, which were treated as artworks. Therefore, it is difficult to publicly include the analyzed data directly in the manuscript/Supplementary material. We will share our data after direct contact with the authors and confirmation of the above restriction. Requests to access these datasets should be directed to Daichi Shimizu, tothefuture0415@yahoo.co.jp.
